# The Effects of a Smartphone App (Feelee) to Enhance Adolescents’ Emotion Regulation Skills in a Forensic Outpatient Setting: Protocol for a Multiple Single-Case Experimental Design

**DOI:** 10.2196/64756

**Published:** 2025-07-02

**Authors:** Merel M L Leijse, Levi van Dam, Thimo M van der Pol, René Breuk, Arne Popma

**Affiliations:** 1 Child and Adolescent Psychiatry & Psychosocial Care Amsterdam UMC Location Vrije Universiteit Amsterdam The Netherlands; 2 Amsterdam Public Health Mental Health Amsterdam The Netherlands; 3 Academic Center for Child and Adolescent Psychiatry Levvel Amsterdam The Netherlands; 4 Dutch Innovation Network for Societal Youth Challenges Garage2020 Amsterdam The Netherlands; 5 Department of Child Development and Education University of Amsterdam Amsterdam The Netherlands; 6 Forensic Mental Health Care Inforsa Amsterdam The Netherlands

**Keywords:** emotion regulation problems, emotional awareness, delinquent behavior, forensic treatment, treatment motivation, smartphone apps, digital phenotyping, single case experimental design, protocol, adolescent, teenager, mHealth, app

## Abstract

**Background:**

Difficulties in emotion regulation are a significant contributing factor to delinquent behavior in adolescence. These adolescents struggle with recognizing, comprehending, and controlling emotions, which impedes the effectiveness of current forensic treatments. In addition, forensic care often faces challenges regarding treatment engagement due to a lack of motivation and difficulties building an alliance between clients and caregivers. The use of Feelee, an app that collects and displays active and passive data, is promising to support adolescents in obtaining more insight into their emotion regulation abilities. Furthermore, the integration of smartphone apps, like Feelee, offers new perspectives to increase adolescents’ engagement and adherence to treatment.

**Objective:**

This study presents the research protocol for evaluating the initial effects of the Feelee app on emotion regulation among adolescents in the forensic outpatient setting. The Feelee app integrates with treatment as usual, and the multiple single-case experimental design methodology is discussed in detail.

**Methods:**

A multiple single-case experimental ABA design was applied to examine the initial effectiveness of Feelee. A total of 24 participants from 2 forensic outpatient care centers completed a 2-week baseline (phase A1), 4-week intervention (phase B), and a 2-week follow-up (phase A2). The primary outcome, emotional regulation, is measured daily using self-reports via the smartphone. Secondary outcomes, including emotional differentiation, insight and self-reflection, emotional awareness, and treatment-related factors such as motivation and therapeutic alliance, are assessed through questionnaires administered at preintervention, postintervention, and follow-up points. Quantitative analyses follow single-case experimental design methods, including visual analysis of individual trajectories, standardized mean difference permutation distance tests, and Cohen *d* at the group level. A 95% CI is calculated per participant to assess change reliability. Secondary outcomes are analyzed using the Reliable Change Index. Qualitative follow-up interviews are analyzed using thematic analysis at both the individual and group levels.

**Results:**

Data collection started in June 2023 and was completed in January 2025. By the time of final manuscript submission, 89 participants had been recruited and 24 had enrolled in the study. Study results will be published in peer-reviewed journals and presented at national and international conferences throughout 2025

**Conclusions:**

This study aims to evaluate the effectiveness of the Feelee app in enhancing emotion regulation skills. By using a multiple single-case experimental ABA design, we will get a first insight into the addition of Feelee to treatment as usual in the forensic outpatient setting. Study strengths include the low-threshold addition, ecological validity, and the use of both quantitative and qualitative research methods. Further implications for clinical practice are discussed.

**Trial Registration:**

Central Committee on Research Involving Human Subjects NL-OMON54390; https://onderzoekmetmensen.nl/en/trial/54390 and ClinicalTrials.gov NCT06509360; https://clinicaltrials.gov/study/NCT06509360

**International Registered Report Identifier (IRRID):**

DERR1-10.2196/64756

## Introduction

### Background

Delinquent behavior in adolescence is associated with the existence of emotion regulation problems [1,2]. As stated in the risk-need-responsibility (RNR) model, these problems arise from various adverse childhood conditions and experiences such as problems in the household (eg, experience of parental divorce, violence, or parental mental illness) or different types of maltreatment (eg, abuse or neglect). The RNR model suggests that adolescents facing these challenges are at a higher risk of engaging in delinquent behavior compared with their typically developing peers [3-6]. Multiple studies show that these harmful experiences negatively impact adolescents’ emotional development [5,7,8]. In particular for emotion regulation, adolescents experience difficulties with recognizing and controlling emotions, culminating in impulsive or aggressive behavior, which increases the risk of showing delinquent behavior [9,10]. Emotion regulation problems affect a variety of negative outcomes such as conflicts at home, school dropouts, or delinquency [11-14]. This highlights the importance of effective emotion-oriented treatments for adolescents to support their emotional regulation abilities and prevent them from future delinquent behavior.

In forensic outpatient youth care, treatment as usual (TAU) consists of both family- and individual-based therapy aimed at addressing the future risk of delinquent behavior. These TAU programs typically integrate elements from cognitive behavioral therapy and regulation skills training, focusing on enhancing cognitive and emotional skills to improve behavioral regulation [15,16]. Although TAU programs are widely considered as evidence-based interventions for prevention and rehabilitation, the effectiveness of these approaches in the forensic outpatient setting has yielded mixed results. For example, multiple meta-analysis found small to moderate effects for family-based treatments results for family-based treatments for the reduction of delinquent or disruptive behavioral problems among adolescents [17-20]. In addition, meta-analyses have shown mixed results on the efficacy of psychosocial and individually oriented cognitive behavioral therapy treatments for reducing disruptive and aggressive behaviors in adolescents. These mixed outcomes can be attributed to differences in the research design, small sample sizes, and limited research on individual treatments in this context [16,21].

The mixed results of TAU programs in the forensic outpatient setting can be attributed to the characteristics of adolescents within this population. A contributing factor is the adolescent’s limited emotional understanding. According to the RNR model, effective interventions should be tailored to adolescents’ risk levels for recidivism, as well as their learning styles and cognitive capabilities [22,23]. However, current TAU requires adolescents to recognize and reflect on their emotions in order to gain insight into their (delinquent) behavior [24,25]. This approach does not align with the cognitive capability of adolescents, who often encounter significant difficulties in maintaining emotional awareness and regulating cognitive responses [26-28]. Additionally, emotion-focused interventions are typically restricted to the treatment setting, neglecting adolescents’ emotional experiences in their everyday lives [29]. This restricted approach hinders a comprehensive understanding of adolescents’ emotional and behavioral patterns, which is critical for accurately assessing and improving their emotion regulation capacities [26,29,30].

Another factor that impedes the effectiveness of forensic outpatient TAU is the lack of motivation and difficulties in building an alliance between adolescents and caregivers, which leads to low engagement and poor adherence to treatment. Among the forensic population, the rates of no-shows and early treatment dropout are notably high: 21.7% among young offenders in sociotherapeutic settings and 57.7% among juvenile inpatient treatments [31]. Additionally, no-show and dropout rates are higher among younger individuals [32]. In most cases, clients have less insight into their problems, a different view of their needs, and are skeptical about the benefits of treatment [33,34]. Establishing a lasting treatment alliance is also challenging due to adverse life events and previous unsuccessful treatment experiences [35]. Noncompletion of treatment significantly increases the risk of recidivism [36]. Consequently, there is a need for supportive tools that align with the perceptions of adolescents in the forensic outpatient setting, in order to enhance their engagement and adherence to TAU [36,37].

The integration of digital phenotyping into TAU could support the enhancement of adolescent emotion regulation skills and increase engagement in forensic outpatient settings. Digital phenotyping involves moment-by-moment quantification through the use of data sources on digital devices (eg, smartphones or smartwatches) to better understand humans’ traits, behavior, and functioning [38,39]. This includes both active data, which requires a short action from the user, and passive data, collected through sensors on the device without requiring a user action [39-41]. Studies have shown promising results regarding the integration of digital phenotyping into clinical practice [42-45]. In treatment, the objectively measured data collected from smartphones provide valuable insights into adolescents’ real-life functioning, including daily emotions and behavioral patterns [45-48]. Furthermore, the use of wearables as part of digital phenotyping enhances adolescents’ self-management, thereby increasing their engagement in treatment [49]. Therefore, integrating digital phenotyping in TAU offers new opportunities to address emotion regulation and motivation problems in the forensic setting.

Feelee is a new smartphone app that leverages digital phenotyping to enhance emotion regulation skills. The app connects digital diary entries by means of emojis (active data) with movement and sleep time (passive data) to provide users with accessible insights into their emotions and behavior. Emojis which include pictographs of faces, objects, and symbols, have been shown to effectively communicate emotions, mood, and physical states [48]. Users are notified 3 times a day (8 AM, 12 AM, and 8 PM, but the times can be adjusted) to enter an emoji. Along with the emoji, users are asked to reflect on 3 questions: “How come you feel this way?” “What are you doing?” and “Who are you with?” Feelee asks to reflect for a moment on the specified emoji. In addition to these active data inputs, Feelee combines the emoji data with passive data on users’ movement and sleep. Poor movement and sleep quality in adolescence are associated with negative emotions and mood disturbances [50-52]. Furthermore, monitored changes in movement and sleep patterns are a strong indicator of the risk of emotion regulation problems [44,53,54]. By combining active emoji and passive behavioral data, Feelee offers valuable insights into adolescents’ daily emotional and behavioral functioning, as well as potential risk patterns in emotion regulation. The low-threshold approach of the app, along with its focus on enhancing emotional regulation skills, makes it a promising transdiagnostic tool for supporting current TAU in forensic outpatient care.

The integration of Feelee into TAU in the forensic outpatient setting can be valuable in 3 aspects of enhancing emotion regulation skills: emotional recognition, reflection, and the development of strategies to manage emotional impulses. First, Feelee facilitates emotional recognition by using preselected emojis, which encourage users to identify and differentiate their emotional states. In TAU, discussions surrounding these emoji selections further support this process, helping adolescents improve their ability to recognize and articulate their emotions [55-58]. This approach also contributes to the development of reflective skills, as both the reflective questions posed by Feelee and the subsequent discussions in TAU further train adolescents’ emotional reflection capacities. Additionally, the weekly overview provided by Feelee, which includes both emoji data and passive behavioral data (eg, physical activity and sleep patterns), supports clinicians and adolescents in identifying patterns and discussing them during treatment sessions. By training individual reflective skills, adolescents gain better insight into their emotional and behavioral functioning (such as physical activities and hours of sleep), which are important aspects of emotion regulation [29,59]. These reflective insights support the development of strategies to manage emotional impulses [60]. Furthermore, enhanced insights into emotional functioning contribute to a better understanding and control of emotional and physical cues, which is considered indicative of improved emotion regulation [60].

Besides emotion regulation, the integration of smartphone apps, like Feelee, offers new perspectives to increase adolescents’ engagement and adherence to treatment. For example, the use of Feelee in treatment better aligns with adolescents’ contemporary interests in digital devices than the usual paperwork (eg, questionnaires or emotion journals) used in mental health care [61]. For adolescents, it is more appealing to be engaged with personalized real-time data that is presented in a visual way [62,63]. Furthermore, the integration of Feelee in treatments contributes to more shared goals, tasks, and decision-making processes, which positively affect the therapeutic alliance [64]. Beyond treatment, Feelee offers opportunities to motivate self-management behaviors by lowering barriers for adolescents to practice emotion regulation skills outside the treatment [48,65]. Hence, Feelee can positively contribute to the adolescents’ motivation and therapeutic alliance, leading to increased engagement and adherence to treatment.

### Study Aims

Given the potential of digital phenotyping to address the need for emotion-focused interventions for adolescents in forensic settings, we previously conducted a feasibility and usability study with a research version of Feelee in addition to TAU [58]. Feedback from participating adolescents and clinicians was incorporated into the development of a new version of the Feelee app. The aim of this article is to provide a detailed description of a multiple single-case experimental design (SCED). The primary objective of this research project is to explore the initial effectiveness of a new version of the Feelee app in addition to TAU to enhance emotion regulation skills. By conducting a SCED, we aim to explore individual changes and between-participant differences on 3 aspects of emotion regulation: recognizing reflection, and the ability to manage emotional impulses. We hypothesize that adolescents will show improvements in the 3 aspects of emotion regulation through the combined use of the Feelee app in TAU; however, we do expect differences in the strength of these improvements between participants.

Secondary objectives include examining the underlying mechanisms through which the Feelee app, as an adjunct to TAU, facilitates the development of emotional differentiation, self-reflection, insight, and emotional awareness. In this context, we also aim to assess the interindividual variability in the effects of the app. We hypothesize that the extent to which the Feelee app contributes to these 3 aspects of emotion regulation will differ across participants. Additionally, we aim to investigate how the use of Feelee to TAU influences shared-goal, decision-making, and self-management within treatment. We hypothesize that the integration of Feelee into TAU will positively affect both treatment alliance and treatment motivation.

The fourth and final objective is to gain an in-depth understanding of the experiences of both adolescents and clinicians using the Feelee app, as well as its application in treatment. We expect that both adolescents and clinicians will respond positively to the use of the Feelee app and will provide valuable recommendations for its further development and integration into treatment.

## Methods

### Study Design

In this study, we apply a multiple single-case experimental ABA design across single participants [66-68]. The multiple ABA-design is a SCED method used to evaluate the effectiveness of an intervention at the individual level [68]. Unlike group-based comparison methods, such as randomized controlled trials (RCTs), which compare outcomes across different groups, we conduct repeated measurements within the same individual under varying conditions [69]. For ABA-designs, repeated measurements are conducted in the absence (A) and presence (B) of an intervention [67,68,70]. This approach allows participants to serve as their own controls [68,69]. Performing a SCED, such as an ABA design, is particularly effective for identifying causal relationships between an intervention (independent variable) and changes in the outcome measures (dependent variables) [69]. Moreover, SCED is an idiographic research method, enabling the tailoring of interventions to the unique needs of participants during the study [71,72]. This makes SCED especially suited for small, heterogeneous populations, where recruiting large numbers of participants can be challenging and time-consuming. This study aims to examine the effects of Feelee in forensic care, which can be characterized as a hard-to-reach study population, making this study design highly suitable. Additionally, the intensive study of a small population in a clinical setting contributes to a better understanding of the potential effects of the intervention within the variability of individuals in the clinical setting [73].

**Figure 1:**
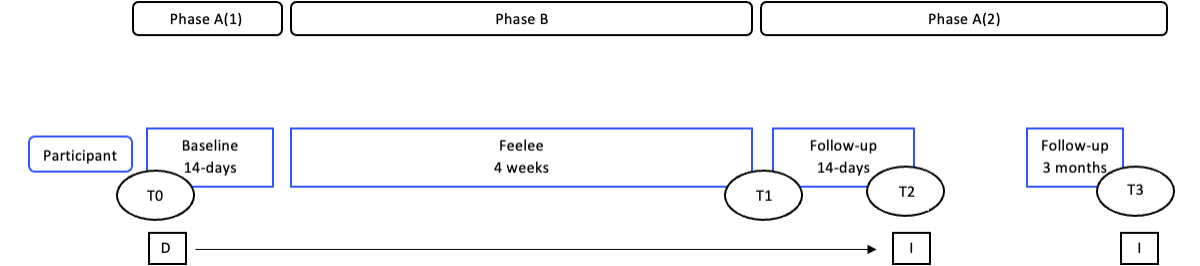
Overview of the study design. Daily repeated measure starts immediately after T0. T1 is conducted on the first day following the 4-week intervention phase. T2 takes place after 2-week follow-up. T3 is conducted 3-months after T2. A(1): baseline; A(2): follow-up; B: Intervention; D: daily questionnaires; I: semistructured interviews; T0: premeasurement; T1: postmeasurement; T2: follow-up measurement; T3: 3-months follow-up measurement.

Participants follow a 2-week baseline phase, 4-week intervention phase, and a 2-week follow-up phase. The inclusion of a baseline phase and the absence of randomization are due to practical considerations related to the hard-to-reach population in this study and the limited effectiveness of randomization for ensuring reliability in this case. Therefore, the phases are kept as short as possible to minimize potential dropout. Primary outcome measurements are taken daily throughout the baseline (phase A1), intervention (phase B), and follow-up phase (phase A2), using the smartphone app M-path. Secondary outcomes are measured at pre-(T0), post-(T1), and short-term follow-up measurements (T2). Additionally, qualitative data collection occurs at T2 and T3. The total research period, from the start of the baseline until the final measurement of the follow-up phase, lasts approximately 8 weeks per participant. A short-term follow-up measurement is taken 3 months after T3. An overview of the study design is presented in Figure 1.

### Participants

Participants are recruited among clients from forensic outpatient teams at Levvel and Inforsa. Both organizations provide mental health care and support for residents of the metropolitan region of Amsterdam. These clients are referred for treatment by a probation officer or general practitioner due to concerns about delinquent behavior and recidivism. The population at Inforsa is slightly older (age 16-27 years) than at Levvel (age 15-21 years). Clients from Inforsa typically have a longer criminal justice history, and most have already been treated in the adult criminal justice system. Since the goal of the Feelee app is transdiagnostic and it can integrate into TAU with minimal barriers and low risks, no distinction is made between types of treatment for participants. However, clinicians are trained to assess the appropriateness of the Feelee app for potential participants, based on the following inclusion and exclusion criteria: (1) aged between 12 and 23 years, (2) at the moment of study in treatment at Levvel or Inforsa, (3) expected to be in treatment for at least 3 more months, (4) own an IOS or Android operating smartphone and (5) have a basic understanding of smartphone use. Exclusion criteria include (1) serious psychiatric conditions, such as psychosis or high risk of suicide, (2) insufficient understanding of the spoken and written Dutch language, and (3) not owning a smartphone.

### Sample Size

The SCED research standards require studies to make at least 3 attempts to demonstrate an intervention effect. Additionally, each phase must include a minimum of 5 data points to effectively demonstrate an effect [70,74]. This means that at least 3 participants must complete a minimum of 5 measurements during the 2-week baseline, 4-week intervention, and 2-week follow-up phase to fulfill these requirements. Therefore, to gain a better understanding of individual differences and enable within- and between-participants comparisons, at least 9 to 10 participants will be included in the data analyses [67].

In addition to the SCED research requirements, the considerable risk for treatment attrition in the forensic population must also be considered. Studies on adolescents’ outpatient mental health care report attrition rates ranging between 28% and 75% [75]. For the forensic population, a meta-analysis on juvenile inpatient treatments shows that dropout rates can go up to 60% [31,32]. To account for a potential 60% attrition rate and to ensure the inclusion of at least 9 participants for demonstrating intervention effects and conducting within- and between-participant analysis, a total of 24 participants will be included in this study.

### Procedures

First, all adolescents in the caseload at the forensic outpatient teams are screened by clinicians. During this screening process, the clinician makes an initial informed assessment of whether the adolescent is eligible to participate in the study. All clinicians are thoroughly briefed about the study design and population in advance (see also section: Feelee training). Additionally, clinicians can always consult the first author or independent experts if they are uncertain about whether to approach an adolescent for the study. This screening method is necessary due to the high risk of nonresponse in the study population. When an adolescent is deemed eligible, the clinician briefly introduces the study to the adolescent. An information handout about the Feelee app and the study will be available for this purpose. If the adolescent meets these criteria and agrees to participate, the researcher arranges an informed consent appointment. During this appointment, the researcher explained the Feelee app and study procedures. The inclusion and exclusion criteria are reviewed again to confirm the adolescent’s eligibility. If the adolescent meets the criteria and agrees to participate, informed consent is signed. If the adolescent is under 16 years old, parental or legal guardian consent is also required, and they need to sign the informed consent form.

After the informed consent procedure, premeasurement is conducted. Following this, daily measurements are set up by installing a data collection app (M-path) on the adolescent’s smartphone. The researcher explains how to use the app and tests it directly. From that moment, adolescents receive a daily notification from the app to complete the measurement within a set time frame. The daily measurement continues throughout the 2-week baseline phase, the following 4-week intervention phase, until the last day of the 2-week follow-up phase. After the 2-week baseline phase, participants start using the Feelee app. The researcher provides them with the login details. From then on, participants select an emoji at least once a day (active data) and allow the app to collect data on their daily steps and hours of sleep per day through sensors (passive data). The clinician reviews the Feelee data during weekly treatment sessions. After the 4-week intervention phase, participants uninstall the Feelee app from their smartphones. Additionally, the researcher schedules an appointment for the postmeasurement. During the 2-week follow-up phase, participants continue completing the daily questionnaire but no longer use the Feelee app. anymore. Thereafter, an appointment is scheduled for the follow-up measurement and semistructured interview. To minimize the effort for participants, both the follow-up measurement and the semistructured interview are conducted in a single appointment. The researcher also arranges a final appointment with the clinician for an interview. Once these steps are completed, the study period closes. Three months after the study ends, participants are asked if they are willing to participate in the 3-month follow-up. If they agree, the researcher schedules the 3-month follow-up measurement and interview.

Participants receive compensation for each completed questionnaire and submitted emoji. The researcher and participant agree in advance on how often participants want to receive an update about the compensation. Additionally, the researcher, participant, and clinician make agreements about the contact moments during the study. The frequency of these contact moments is kept as consistent as possible among participants. However, it may vary slightly between participants, for example, to encourage the daily measurements. All contact moments are logged to track variations, which will be considered during the analysis.

### Feelee App Use

**Figure 2:**
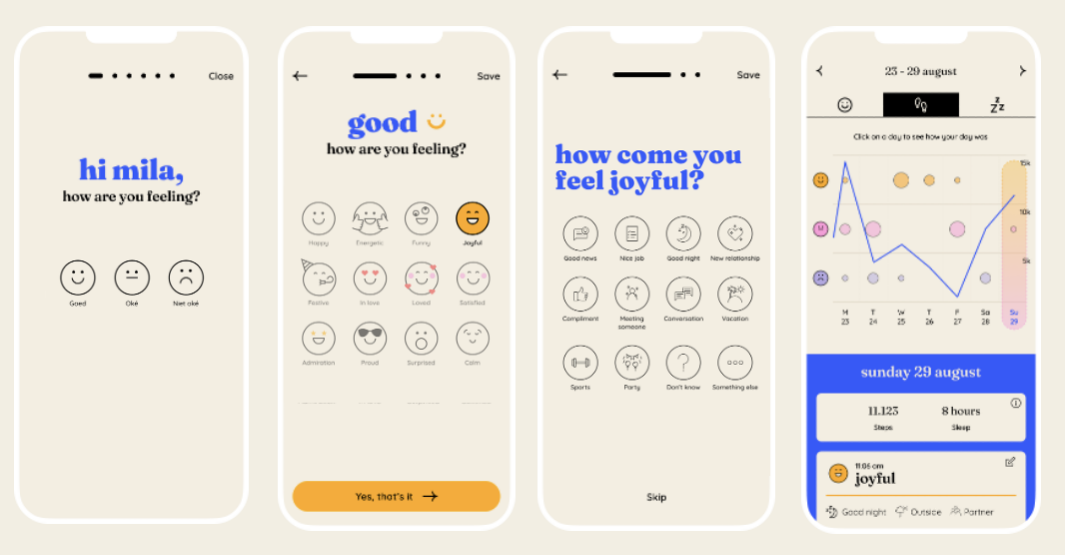
Screenshots from the Feelee app.

The use of the Feelee app in this study is twofold. First, participants use the app independently, submitting at least one emoji each day and granting Feelee permission to read data on the number of steps and hours of sleep. After each emoji submission, participants receive advice that could positively influence emotions in daily life or a question to reflect on emotions and behavior. Furthermore, Feelee provides a weekly overview of the submitted emoji and collected behavioral data that can be viewed at any time (Figure 2).

Second, the weekly overview is discussed during weekly individual TAU sessions. In this process, clinicians follow a number of steps that contribute to the adolescents’ emotional understanding [58]. Step 1 involves emotion recognition. Clinicians select one emoji from the weekly overview and ask the participant questions in order to feel and recognize the emoji in their body. Step 2 focuses on reflection. When submitting an emoji, Feelee forces participants to reflect on their emoji with a few short questions. In this step, participants and clinicians review the answers together and discuss them further. The most important aspect of this step is that participants can recall which circumstances in their daily lives may have influenced their emotions. Step 3 involves understanding patterns in emotions. In this step, clinicians analyze the Feelee data to identify patterns, such as whether certain emojis are linked to specific days of the week, activities, or social contacts. These questions help participants gain insight into the contextual and behavioral factors that may influence their emotions. Last, step 4 involves examining patterns in activity and sleep data alongside the emojis. Both participants and clinicians explore how the activity and sleep data might influence the emojis selected by the participant.

### Feelee Training

All clinicians in the participating teams complete a 2-day training session conducted by the first author, developed in collaboration with the third and fourth authors. On day 1, clinicians receive background information on the development of the Feelee app and study objectives. Between the first and second day of training is at least 14 days, during which clinicians are instructed to use the Feelee app by themselves. On day 2, clinicians exchange their experiences with the app and receive guidance on how to integrate Feelee and its data into treatment. This includes instructions on interpreting the app’s data as well as identifying and addressing relevant patterns. These instructions are informed by the Feelee manual, specifically developed for the forensic population, which is distributed to clinicians during the training. Clinicians also participate in a role-play exercise based on the manual, providing them with an opportunity to practice using the Feelee data in a clinical context. Additionally, clinicians are briefed on the participant selection criteria, ensuring they understand the process for identifying eligible participants and the potential risks associated with participation in the study. Throughout the research period, clinicians received ongoing guidance through different contact moments with the first author. To assess adherence to the Feelee manual and study protocol, clinicians complete a brief questionnaire following each treatment session during the intervention phase.

### Measures

#### Primary Outcome Measure

##### Assessment

The primary outcome measure is collected daily through a short self-report questionnaire on the adolescents’ smartphones. Participants received a notification on their smartphones after 3 PM to complete the questionnaire before the end of the day.

##### Emotion Regulation

Emotion regulation is measured using a short composite questionnaire comprising items from the Difficulties in Emotional Regulation Scale (DERS-36) and Regulation of Emotion Systems Survey for Ecological Momentary Assessment [76-78]. The questionnaire includes 2 items from the DERS-36 that focus on recognizing and managing emotions, adapted for daily use. Answers to the DERS-36 can be given by a 5-point Likert-scale from “strongly disagree” to “strongly agree” [77]. Additionally, 4 items from the Regulation of Emotion Systems Survey for Ecological Momentary Assessment, representing rumination, reappraisal, distraction, and suppression, are included to capture the various aspects of emotion regulation strategies. Answers can be given from 0=“not at all” to 10=“very much” [78].

#### Secondary Outcome Measures

##### Assessment

The secondary outcome measures include the potential working mechanisms of the Feelee app (emotional differentiation, reflection, and awareness), as well as treatment-related factors such as motivation and alliance. These measures are collected at pre-(T0), post-(T1), and follow up-(T2) measurements.

##### Emotional Differentiation

Emotional differentiation is measured using the Positive and Negative Affect Schedule [79]. The Positive and Negative Affect Schedule asks participants to rate 20 items of prewritten emotions, including 10 positive and 10 negative emotions, presented in random order. The items are scored on a Likert scale ranging from “Very slightly/not at all” to “Extremely.” Both the positive affection and negative affection scales demonstrate high reliability [79].

##### Emotional Reflection

Emotional reflection is assessed using the Self-reflection and Insight Scale for Youth (SRIS-Y) [80]. The SRIS-Y is a 17-item self-report questionnaire, answered on a 6-point Likert scale ranging from “disagree strongly” to “agree strongly.” The instrument consists of 2 subscales, self-reflection and insight, which provide separate subscores. The original adult version has been shown to be a reliable and valid measure of self-reflection and insight [81], and the SRIS-Y is considered a reliable tool for measuring these constructs in adolescents [80].

##### Emotional Awareness

Emotional awareness is measured by the Multidimensional Assessment of Interceptive Awareness [82]. The Multidimensional Assessment of Interceptive Awareness includes 32 items across 8 different subscales: noticing, not-distracting, not-worrying, attention regulation, emotional awareness, self-regulation, body-listening, and trusting. Answers can be given on a 6-point Likert scale ranging from “never” to “always.” To minimize the questionnaire length, only the Emotional awareness subscale is used, which demonstrates good reliability [82].

##### Treatment Motivation

To measure the participant’s motivation for treatment, the Dutch Adolescent Treatment Motivation Questionnaire (ATMQ) is used [83]. The ATMQ consists of 11 self-report items rated on a 3-point Likert scale, including “not true,” “kind of true,” and “true.” Overall, the ATMQ demonstrates good reliability [83].

##### Treatment Alliance

Treatment alliance is assessed using an abbreviated Dutch translation of the Working Alliance Inventory (WAV-12) [84]. The questionnaire includes versions for youth, parents, mentors, and family therapists, with only the youth version used in this study. The WAV-12 consists of 12 self-report items rated on a 5-point Likert scale from “rarely or never” to “always.” Higher scores on this questionnaire indicate a stronger working alliance between the client and clinician. The Dutch version of the WAV-12 showed high reliability [84].

### Other Study Parameters

#### Sociodemographic Parameters

The following social demographic information is collected: age, sex, native country, language, second language (optional), education level, ethnicity, living situation, marital status of parents, and possible experiences with delinquent behavior. The information is gathered through a demographic questionnaire developed by the researchers. Furthermore, background information about the treatment process, such as diagnostic background and treatment history, is obtained from file information provided by the clinician.

#### Treatment Integrity

Treatment integrity is intended to briefly capture the use of the Feelee app during treatment sessions. It is measured through a short questionnaire completed by the clinician after each weekly treatment session during the intervention phase (phase B). The questionnaire includes 4 short questions regarding the availability of data in the Feelee app and whether these data were discussed during the treatment session.

#### Feelee App Parameters

The following study parameters are derived from the use of Feelee app during the 4-week intervention phase.

#### Emotional Well-Being

Emotional well-being is measured by active monitoring. During the intervention phase, participants complete a short emoji questionnaire in the Feelee app once a day. The selected emojis are presented in a weekly overview in the Feelee app.

#### Physical Activity

Insight into physical activity is obtained through passive monitoring, with data read from the health app (Google Fit for Android, Health app for IOS) by the Feelee app. Physical activity is measured by the number of steps taken each day, tracked by a pedometer on a smartphone. The Feelee app provides a weekly overview of the daily step count, displayed alongside the selected emoji.

#### Sleep Activity

Sleep activity is also monitored passively through data collected by the health app. It is measured by the reported bedtime in combination with screen time and movement (via the pedometer). The Feelee app provides a weekly overview of the hours of sleep per night, displayed both with and without the associated emojis.

### Evaluation

#### Assessment

During the follow-up phase, both quantitative and qualitative evaluations are conducted to assess overall experiences regarding the Feelee app and the use of the smartphone app during treatment.

#### Evaluation Feelee App

To evaluate the Feelee app, we first conduct 2 short questionnaires: the Twente Engagement with eHealth Technologies Scales [85] and the System Usability Scale [86]. The Twente Engagement with eHealth Technologies Scale consists of 9 items about engagement, of which 3 assess behavioral engagement, 3 assess cognitive engagement, and 3 assess affective engagement. Answers are given on a 5-point Likert scale from “strongly disagree” to “strongly agree.” The overall questionnaire shows good reliability [85]. In addition, the System Usability Scale is used as a quick tool to measure the usability of Feelee. A total of 10 items can be answered on a 5-point Likert scale from “strongly disagree” to “strongly agree” [86]. The questionnaire also shows good reliability [87].

To get an in-depth understanding of the use of Feelee, semistructured interviews with both adolescents and clinicians are conducted. The interviews focus on experiences regarding emotional skills and changes in treatment-related factors (motivation and treatment alliance). Adolescents and clinicians are asked about possible changes during treatment sessions and whether these changes may be attributed to the use of Feelee. The interviews provide opportunities to get a deeper insight into the adolescents’ and clinicians’ experiences, as well as feedback for further improvements in the use of the Feelee app.

#### Evaluation Smartphone Use in Treatment

In addition to the evaluation of the Feelee app, questions are added to the interviews about the general use of smartphone apps and smartphone data in treatments. Adolescents and clinicians are asked to reflect on their experiences regarding the use of smartphones in treatment and whether they liked working with them. Further, special attention is paid to the use of smartphone data (active and passive) during the treatment session.

**Figure 3:**
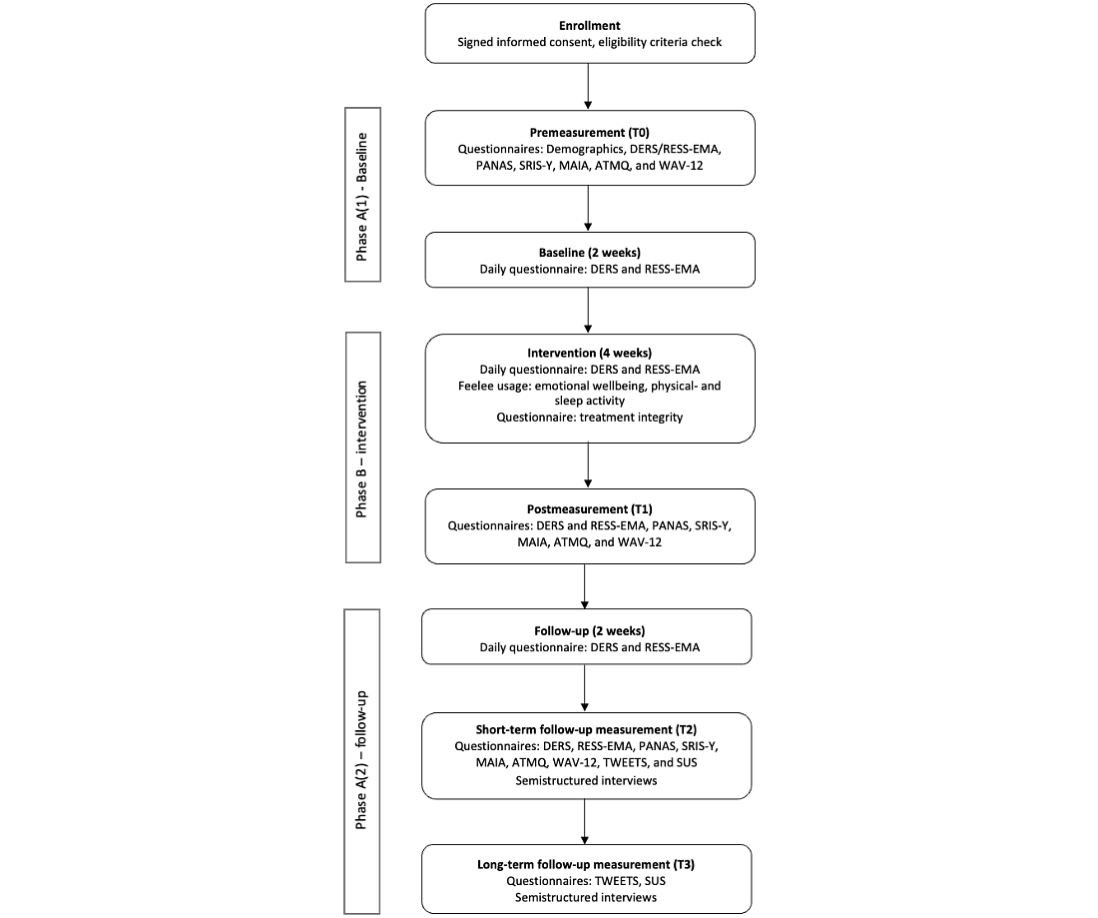
Overview data collection moments. ATMQ: adolescent treatment motivation questionnaire; DERS: Difficulties in Emotion Regulation Scale; MAIA: multidimensional assessment of interceptive awareness; PANAS: Positive and Negative Affect Schedule; RESS-EMA: Regulation of Emotion Systems Survey for Ecological Momentary Assessment; SRIS-Y: Self-reflection Insight Scale for Youth; SUS: System Usability Scale; TWEETS: Twente Engagement with eHealth Technologies Scale; WAV-12: Working Alliance Inventory.

Figure 3 and Table 1 present an overview of all measurements and data collection moments.

**Table 1:** Overview variables, measurements, and informants.

Variable			Measure	Measuring point	Informant
**Primary outcome**					
	Emotion regulation		DERS^a^, RESS-EMA^b^	Daily questionnaire	Adolescent
**Secondary outcomes**					
	Emotional differentiation		PANAS^c^	T0, T1, and T2	Adolescent
	Emotional reflection		SRIS-Y^d^	T0, T1, and T2	Adolescent
	Emotional awareness		MAIA^e^	T0, T1, and T2	Adolescent
	Treatment motivation		ATMQ^f^	T0, T1, and T2	Adolescent
	Treatment alliance		WAV-12^g^	T0, T1, and T2	Adolescent
**Other study outcomes**					
		Demographics	Demographic questionnaire	T0	Adolescent
		Diagnostic and treatment history	File information	T0	Clinician
		Emotional wellbeing	Feelee app	Intervention phase	Adolescent
		Physical activity	Feelee app	Intervention phase	Adolescent
		Sleep activity	Feelee app	Intervention phase	Adolescent
		Treatment integrity	Questionnaire	Intervention phase	Clinician
		Evaluation Feelee app	TWEETS^h^, SUS^i^, and semistructured interviews	T2 and T3	Adolescents and clinician
		Evaluation smartphone use	Semistructured interviews	T2 and T3	Adolescent

^a^DERS: Difficulties in Emotion Regulation Scale.

^b^RESS-EMA: Regulation of Emotion Systems Survey for Ecological Momentary Assessment.

^c^PANAS: Positive and Negative Affect Schedule.

^d^SRIS-Y: Self-reflection and Insight Scale for Youth.

^e^MAIA: Multidimensional Assessment of Interceptive Awareness.

^f^ATMQ: Adolescent Treatment Motivation Questionnaire.

^g^WAV-12: Working Alliance Inventory.

^h^TWEETS: Twente Engagement with eHealth Technologies Scale.

^i^SUS: System Usability Scale.

### Analysis

The primary outcome measure, emotion regulation, is assessed daily through a self-report questionnaire completed on the participants’ smartphones. As prescribed by SCED-methods, results are analyzed using visual analyses [68,74]. For this purpose, the hypotheses for each item are plotted visually, mapping the expected direction and strength of change between phases. Graphical visualizations are conducted at both the within-individual participant and between-participant levels. Through visual inspection, the degree of spread between the daily measurements, the potential rate of change across all phases, and the degree of change regarding the hypothesis are examined for each participant individually. To assess the reliability of potential changes, a 95% CI is calculated for each participant using standard errors of difference. For each item, a standardized mean difference permutation distance test is performed for both each participant and the group level, with a *P* value of <.05 considered significant. [88]. At the group level, we also perform a Cohen *d* test. If necessary, additional analyses related to the SCED-procedure will be conducted

The secondary outcome measures are collected during pre-(T0), post (T1), and follow-up measurements (T2 and T3) and semistructured interviews during both follow-up measurements. For the quantitative data, each data point is analyzed by computing a Reliable Change Index (RCI) [89]. The RCI is used to assess whether the observed change in an individual’s score is statistically significant and exceeds the potential measurement error. A change is considered reliable if the RCI meets the criteria of >1.96. The RCI scores of participants are compared with the results from the primary outcome.

For the qualitative data, interviews are recorded and transcribed. The anonymous transcripts are then analyzed at both the individual and group levels. At the individual level, the analysis aims to identify explanations underpinning each participant’s quantitative results. At the group level, recurring themes and shared experiences across participants are examined to identify overarching patterns. For both levels of analysis, reflexive thematic analysis [90] is performed using MAXQDA [91]. The coding process begins with an initial phase at the individual level, where codes are defined based on participants’ quantitative data, including experienced effects on the emotion variables, treatment factors, and evaluation of Feelee app use. These codes are iteratively refined and expanded following a thorough review of the transcript from both the participant and the involved clinician. Subsequently, a separate coding phase is conducted at the group level to systematically identify patterns and themes across participants. All transcripts are independently coded by 2 researchers. After each round of data analysis, discussions are held to ensure consensus on the identified codes for individual participants and consistency in the identification of group-level themes. To enhance transparency and maintain methodological rigor, each researcher keeps a logbook documenting the analysis process.

### Ethical Considerations

This study was approved in April 2023 by the independent Medical Ethical Committee of Vrije Universiteit Medical Center (reference number: 2022.0398). Since this study involves clinical research that includes the smartphone in treatment, the Medical Device Regulation is applicable [92]. Therefore, in addition to the ethical aspects, the study is also reviewed on the technical functioning and safety. No participants younger than 12 years were included in this study. During informed consent, adolescents give explicit permission for the audio recording of interviews and for the collection of both active and passive data by the Feelee app. Audio recordings are used to produce deidentified transcripts of the interviews and are permanently deleted immediately after transcription. Both the recordings and transcripts are securely stored on an encrypted hard drive within the research department. Further details on data handling and storage, including those related to the Feelee app, are provided in the section “Confidentiality of Data.” Participants received a gift voucher of up to €33 (US $38.66) as compensation for their contribution, depending on their level of engagement with both the questionnaires and the daily emoji submissions in the Feelee app. This manuscript does not contain any identifiable images or data related to participants. Further implementation of the study proceeds according to the principles of the World Medical Association Declaration of Helsinki, the Medical Research Involving Human Subjects Act, and guidelines of the Medical Device Regulation [93].

### Confidentiality of Data

Handling and storage of data are done in accordance with the General Data Protection Regulation. Furthermore, a data protection impact assessment and confidentiality, integrity, and availability are performed in collaboration with the privacy officer from Amsterdam UMC to identify potential risks and measures for data collection and storage. Study data, including questionnaires and interviews, are collected by researchers from the Department of Child and Adolescent Psychiatry at the Amsterdam UMC. All data are collected and stored under a unique participant ID number. For the daily questionnaire, data are stored in a protected folder from the M-Path app on the participant’s smartphone. This folder is only accessible by M-Path. Furthermore, the storage data in M-Path consists only of bytes, which means the content of the data is not visible. At the end of the study, the M-Path data are exported to the participant folder that is kept on the secure disk of the study project in the department. Data from the other questionnaires are collected and stored on the Castor Electronic Data Capture database. During the intervention, participants use the Feelee app. Since users of Feelee normally log in with their private email addresses, participants in this study will receive a unique email address and password. In this way, Feelee’s data from participants remains unidentified. Data from Feelee is stored on a secure server managed by a professional ISO 27001 qualified hosting organization. At the end of the study, a data export of the Feelee data is conducted to match participants’ Feelee data with the participants’ measurements in the intervention phase. Last, interviews are recorded by a recording device. After the transcript of these recordings is completed, the recordings are deleted. In accordance with the guidance from the Board of Directors of the Amsterdam UMC, research data and analyses are stored for 15 years after finishing the research project.

## Results

Data collection started in June 2023 and was completed in January 2025. At the time of final manuscript submission, 89 participants had been recruited, 24 had enrolled, and 15 had been retained in the study. Data analysis started after the final data collection had been completed. Study results will be published in scientific peer-reviewed journals and presented during international and national conferences. The study has been registered at the medical ethics committee of the Vrije Universiteit Medical Center, the Netherlands (2022.0398/NL78889.029.21) and at the ClinicalTrials.gov registry (NCT06509360)

## Discussion

### Study Aims

While current treatments in the forensic outpatient setting are often limited by providing sufficient insight into daily emotion and behavioral functioning, as well as motivating and engaging adolescents for treatment, Feelee offers new opportunities to meet these challenges [48,58]. In this study, we elaborated the protocol of the SCED study in order to conduct an initial exploration of the effectiveness of Feelee in forensic outpatient TAU. By using both quantitative and qualitative methods, we are able to get an in-depth understanding of the individual effectiveness and experiences of Feelee in a clinical setting. Thereby, we aim to conduct an adequate and effective innovative tool to support the treatment of adolescents with emotion regulation problems.

### Strengths and Challenges

The study has multiple strengths. First, in order to explore the initial effects, a SCED will be applied instead of more traditional group comparison designs, such as an RCT. Besides the practical advantages (eg, less time-consuming and more cost-efficacy), SCED provides higher internal validity than traditional group comparison designs [68,74]. Throughout a SCED baseline, it is possible to collect a large amount of individual and contextual information within the entire study process while respecting the personal variability of each participant [74]. Additionally, it is possible to include the real-time monitored data from Feelee to increase the number of observations that capture a more accurate presentation of the outcomes on the individual level [94]. This provides a lot of insights about the individual trajectory of the intervention for each participant, which allows us to get an in-depth understanding of the circumstances under which Feelee could be supportive in their treatment. Second, through the use of repeated measurement within and beyond treatment sessions, we are able to get insight into the real-life circumstances of the use of Feelee in addition to treatment. This provides a unique insight into the specific needs of participants, allowing better-tailored care [95]. Furthermore, these insights contribute to strengthening the ecological validity of the study [67,71,96]. Last, the use of Feelee has been applied and studied in TAU, which means participation in this study is known for its low threshold. Therefore, participating in the study poses a minor risk and burden for participants.

In addition to the strengths, this study also faces several potential challenges. First, as described in this study, treatments in the forensic setting are typified by a high number of nonresponses and dropouts [31,32]. Despite several measures being taken (eg, keeping the study design and questionnaires as short as possible), nonresponse and dropout will still be conceivable. Therefore, the research team will try to facilitate the participants as much as possible. This may include scheduling appointments at the participant’s treatment location, but also staying in touch with participants during the study by sending reminders for appointments or questionnaires. Second, another issue related to the risk of nonresponse is the potential for selection bias. Participants are recruited through preselection by the researchers in consultation with the involved clinicians. This approach risks excluding adolescents who are less motivated for treatment and belong to a higher risk group for recidivism. Therefore, the training for clinicians emphasized the potential risk of selection bias. In addition, researchers will continue to emphasize the risk in the discussions with clinicians to ensure appropriate considerations will be made during recruitment. Third, this study makes use of validated self-report questionnaires, which potentially causes the risk of social desirability bias. However, a mixed method approach will be applied to combine the qualitative outcomes with quantitative results. This triangulation contributes to the interpretation of the study results and reduces the limitations of self-reporting. Last, Feelee is a new innovative tool that is still in the development phase, meaning the potential risks and benefits have yet to be fully established. As such, during clinician training, particular attention is given to the potential risks associated with participant involvement in this study, ensuring these factors are carefully considered during participant selection. Moreover, studying the integration of Feelee in treatment requires a cautious and precise research approach. Therefore, the use of a SCED rather than an RCT is particularly suitable at this stage of development and research [66,67].

### Implications for Practice

Feelee is a new innovative smartphone app that could be valuable in improving adolescent emotion regulation skills and contribute positively to treatment motivation and alliance. The study will demonstrate that, while observing medical-ethical and privacy standards, it is possible to safely use smartphone data in treatment. This contributes to new insights and perspectives in data-driven treatment. In addition, the description of the study protocol will show the possibilities of clinical research for adolescents with delinquent behavior, a population known to be hard to reach. Consequently, this population is often overlooked in research due to the high dropout risk. However, these adolescents, in particular, would benefit from new innovations that better suit their perspectives and interests. Conducting a SCED instead of an RCT contributes to the flexibility required for this population and the achievability of the required sample size. This study shows possibilities for conducting innovative research in difficult clinical environments. As such, the study contributes to new insights in science and clinical practice.

### Conclusions

To date, the development and research of smartphone apps for the forensic population remains limited. The apps that are available are often developed and designed for adults [97], or additional wearables (such as smartwatches) are needed for app use [98]. With respect to improving emotion regulation skills, apps are often limited to tracking daily emotions. In contrast, Feelee tracks and displays both emotions and behavior aspects (eg, movement and sleep). Additionally, the Feelee app is designed for use on smartphones without the need for other wearables, increasing its user-friendliness and accessibility.

So far, only feasibility and usability studies using a research version of the Feelee app have been published [48,58]. However, no empirical research has been conducted to explore the effectiveness of Feelee in clinical treatment. This study will be the first that will explore the value of Feelee in enhancing emotion regulation skills in the treatment of delinquent behavior. Results will contribute to the further development and design of follow-up studies of Feelee in treatment.

## Abbreviations

ATMQ: adolescent treatment motivation questionnaire
DERS: Difficulties in Emotion Regulation Scale
RCI: Reliable Change Index
RCT: randomized controlled trial
RNR: risk-need-responsibility
SCED: single-case experimental design
SRIS-Y: self-reflection insight scale for youth
TAU: treatment as usual
WAV-12: working alliance inventory

## Data Availability

The datasets generated or analyzed during the study, for which this protocol is described, will be available from the corresponding author upon reasonable request, once the research and data analyses have been completed.
